# Noise levels in a dental teaching institute - A matter of concern!

**DOI:** 10.4317/jced.50725

**Published:** 2012-07-01

**Authors:** Simarpreet Singh, Ramandeep S. Gambhir, Gurminder Singh, Sumit Sharma, Amarinder Kaur

**Affiliations:** 1 BDS, MDS. Associate Professor. Dept. of Public Health Dentistry. Gian Sagar Dental College and Hospital, Rajpura, Punjab.; 2 BDS, MDS, MPH. Assistant Professor. Dept. of Public Health Dentistry. Gian Sagar Dental College and Hospital, Rajpura, Punjab.; 3 BDS, MDS. Professor. Dept. of Prosthodontics, Gian Sagar Dental College and Hospital, Rajpura, Punjab.; 4 BDS, MDS. Reader. Dept. of Periodontics. Gian Sagar Dental College and Hospital, Rajpura, Punjab.; 5 BDS, MDS. Assistant Professor. Dept. of Oral Medicine and Radiology. Gian Sagar Dental College and Hospital, Rajpura, Punjab.

## Abstract

Objective: To measure and assess the noise levels produced by various dental equipments in different areas of a dental institution and to recommend improvements if noise levels are not within permissible limits. Material and Methods: Sound levels were measured at three different areas of a dental institution where learning and teaching activities are organized. The sound level was measured using a sound level meter known as ‘decibulolmeter’. In each area the noise level was assessed at two positions-one, at 6 inches from the operators ear and second, at the chairside instrument trolley. Noise levels were also assessed from a central location of the clinic area when multiple equipments were in operation simultaneously. Results: Dental laboratory machine, dental hand-piece, ultrasonic scalers, amalgamators, high speed evacuation, and other items produce noise at different sound levels which is appreciable. The noise levels generated varied between 72.6 dB in pre-clinics and 87.2 dB in prosthesis laboratory. The results are comparable to the results of other studies which are conducted elsewhere. Although the risk to the dentists is lesser, but damage to the hearing is possible over prolonged periods. Conclusion: Higher noise levels are potentially hazardous to the persons working in such environments especially in the laboratory areas where noise levels are exceeding the permissible limits.

** Key words:**Noise level, equipment, hearing loss, risk, working areas.

## Introduction

There are many putative health effects which are produced in the human body because of noise (1). Noise pollution has become a common concern for all the members of the society. The National Institute for Occupational Safety and Health (NIOSH) has identified noise as one of the ten leading causes of work related diseases or injuries. Noise can cause masking of unwanted sounds, interference with speech and communication, pain and injury, and temporary and permanent hearing loss (2). Prolonged exposure to noise can lead to noise-induced hearing loss (NIHL) and it may be undetected for years since it is estimated that individuals lose about 28 % of hearing before becoming aware of the problem (3).

According to reports from Occupational Safety and Health Administration (OSHA) just 8 hours of continual exposure to a noise level of 85 decibels is permissible daily (2). Dentists and dental auxiliaries are exposed to noise of different sound levels while working in dental clinics and laboratories (2,4). Dental laboratory machine, dental hand-piece, ultrasonic scalers, amalgamators, high speed evacuation, and other items produce noise at different sound levels which is appreciable (5). Hearing problems were reported by few dentists in a study (6).

Many investigators have examined noise exposure of dentists and the consequences of this exposure with varying results. According to a study, the laboratory machines produced more noise (81.42 dB) compared to laboratory electromotor (74.95 dB), turbine handpiece (72.91 dB) and low speed handpiece (69.71dB) creating a greater risk to laboratory technicians working for more than 8 hours (2). The results of another study found significant differences in the noise levels at 6m and 2 inches from the operator ear, the laboratory engines producing the highest noise (7). Some authors have proposed that ultrasonic scalers may be a potential hazard to the auditory system of both clinicians and patients (8,9). Several questions have also been raised in a number of studies regarding the impact of dental-handpieces upon hearing health of dentists (10-14).

Hence this study was undertaken to measure and assess the noise levels produced by different dental equipments in a dental institution, to determine if the noise level exceeds health limits and to recommend improvements if noise levels are not within permissible limits.

## Material and Methods

The present study was conducted in the Manipal College of Dental Sciences, Manipal. Prior permission and ethical clearance from the Dean of the institution was obtained for the study. The various dental handpieces and equipment used in various departments and laboratory were checked to measure the level of noise produced by each.

**Figure 1:**
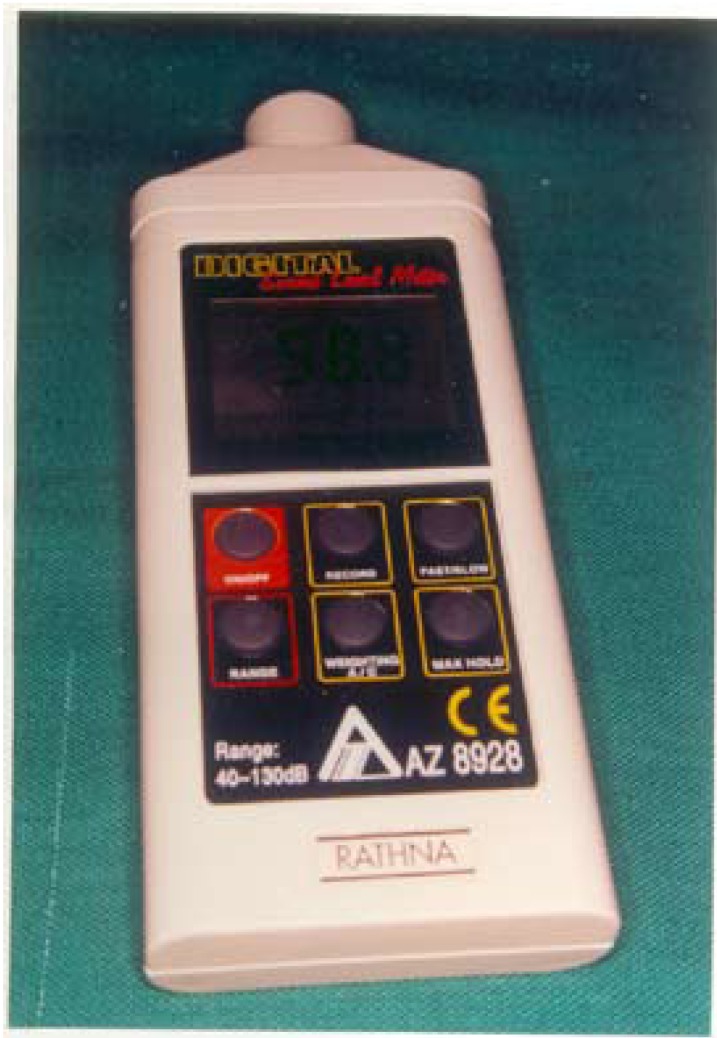
Decibulometer or Sound Level Meter used to measure noise emitted by different dental equipments.

The instrument used to measure the level of noise was a “decibulometer” or a “sound level meter” (Fig. 1) with a mounted microphone directed towards the source of sound in different teaching and learning areas. In each area the noise level was assessed at two positions-one, at 6 inches from the operators ear to simulate the noise reaching the ear drum and second, at the chairside instrument trolley to simulate the noise reaching the person standing nearby, although in decreased intensity. The purpose was to find out the noise levels in the immediate vicinity of the working area of the operator. This standardized instrument was obtained from the Manipal Institute of Technology, Manipal. The investigator was calibrated by training sessions in the correct use of the decibulometer from the concerned personnel of the Manipal Institute of Technology, Manipal. At all times, the decibulometer was used according to manufacturer’s recommendations.

The learning-teaching areas and the activities assessed in this study were the following-

(1) Prosthesis Laboratory- Lab activities involving the use of mounted metal trimmer, acrylic trimmer and sand paper mandrel was assessed. Also assessed was the level of noise produced when lathe gypsum trimmer and lathe acrylic trimmer were used.

(2) Preclinical- Activities mostly comprise the use of hand micro-motor in phantom-head preclinical labs.

(3) Clinics- Activities involving the use of different scalers (sonic, magnetostrictive and peizo) and the use of high speed air-rotors used while cutting tooth structure.

The operating pressure for equipment was the maximum permissible value recommended by the manufacturer. The readings were taken when the suction pump was also in operation along with the equipment. All the hand-pieces and other equipment measured for the noise level were new and in good running condition.

Finally, the data was collected and analyzed using simple statistical representation. Microsoft excel was used to conduct all the necessary statistical calculations. Standard statistical tests were not applied as the study did not aim to correlate the levels of noise to any auditory effects on the personnel in the concerned hospital. Any correlation between the noise levels and the auditory effects would require a detailed evaluation of various parameters related to the personnel’s profile like age, sex, time spent in the operatories each day, type of operatory an individual works in, duration since the exposure to such noise levels started (work experience) etc.

## Results

**Figure 2:**
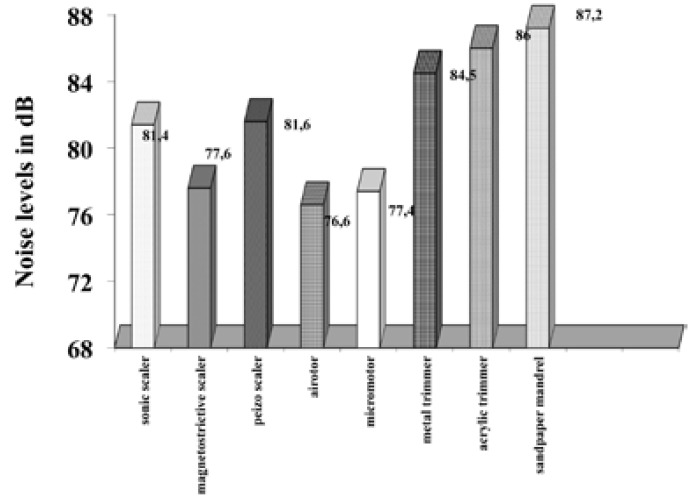
Noise levels of dental equipments measured at 6 inches from the operator’s ear.

**Figure 3:**
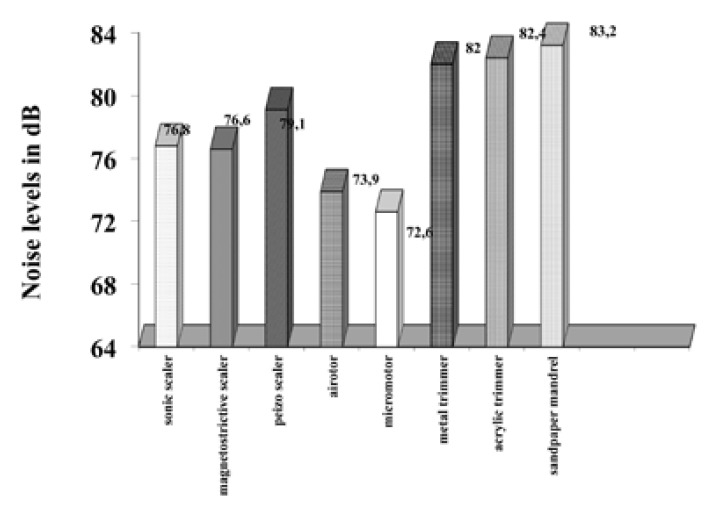
Noise level of dental equipment measured at chairside instrument trolley.

All the dental equipments considered as a potential source of noise pollution were assessed for noise levels with the help of a “decibulometer” or “sound level meter”. The microphone of the decibulometer was directed towards the source of the sound at all times for all measurements. The results obtained measure the noise levels at 6 inches from the operator’s ear (Fig. 2) and at the chair side instrument trolley (Fig. 3). The results displayed in Figure 1 show that the sound levels at 6 inches from the operator’s ear vary from 76.6 dB in the clinic areas to 87.2dB in the Prosthesis laboratory. Also Figure 2 shows the sound levels at the chairside instrument trolley also vary between 72.6 dB in pre-clinics to 83.2 dB in prosthesis laboratory.

Noise levels were also assessed from a central location of the clinic area when multiple scalers [30 in number], air-rotors [30 in number] and micro-motors [70 in number] were used simultaneously in their respective work areas ( Table 1). The results displayed in Table 1 show that noise levels at a central location were 83 dB in when multiple scalers were functioning, 81.4 dB when multiple air-rotors were operated simultaneously and 80.1 dB when multiple micro-motors were functioning.

**Table 1:**
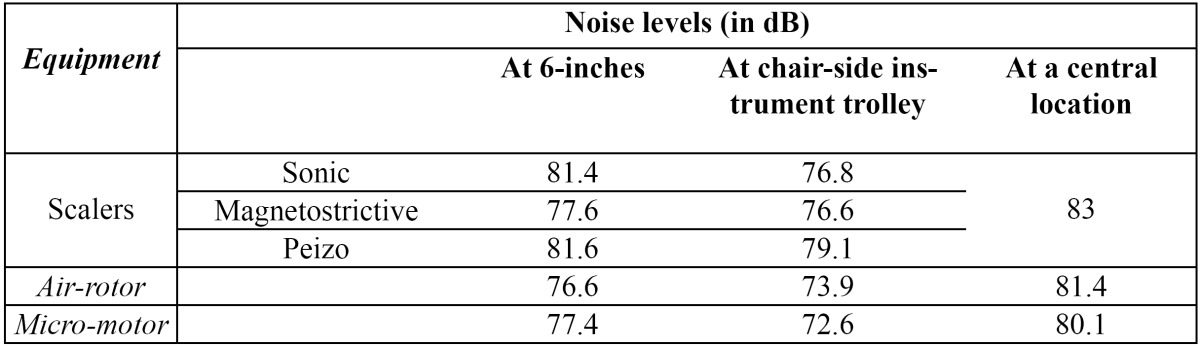
Comparison of noise levels during individual and simultaneous use of equipment.

## Discussion

Noise is a recognized health hazard. High levels of sound are said to be particularly disruptive for the dual task paradigms, requiring attention sharing and sequential responding, involving speed and accuracy. Noise produced in a dental institution where dental handpieces and other equipments are used is a matter of concern. This puts the dental teaching as well as non–teaching staff at potential risk of noise–induced hearing loss (NIHL) (3) which intensifies during life (15). Although this risk can be minimized by successful application of hearing conservation and engineering control (11). Hence this study was conducted to find out if the noise levels produced by various dental equipments were within permissible limits.

The results of this study are comparable to the results of other international studies on noise in dental settings. According to reports of a Finnish study, the noise produced by dental high speed drilling instruments reach the dentist’s ear at 73-85 dB. Similarly, in the present study it has been observed that the noise level from high-speed air-rotor ranged from 73. 9 dB to 81.3 dB (16). Another study conducted on noise levels of various dental handpieces and equipments showed that the maximum noise was produced by laboratory machines with the noise levels reaching up to 85.3 dB which is similar to the findings of this study (2). The sound levels of different equipments like scalers, high speed air-rotors and micro-motors are in accordance to some other studies carried out in some other countries (2,3).

According to OSHA guidelines, just 8 hours of continual exposure to a noise level of 85 dB is permissible daily (2). Typically, dentists do not use high speed handpieces continuously over an 8-hour per day. Most are found to use the high speed handpieces intermittently for 15-30 seconds. This value may indicate the risk of developing noise induced hearing loss (NIHL) solely from dental drills is minimal. Although according to another study, high speed handpieces emit noise at frequencies that may cause hearing loss over time (17). In this study it was observed that when multiple dental units (high speed handpieces and ultrasonic scalers) were used the maximum noise levels recorded were 81.3 dB (high speed handpiece) and 83 dB (scalers). These values may be below the maximum permissible value of 85 dB mentioned by OSHA, yet caution should be used to draw the conclusions because these noise levels have a potential to cause damage over a prolonged exposure.

The noise level recorded from the dental laboratory was found to exceed the maximum permissible value of 85 dB. This is a matter of concern as it puts the dental technicians at high risk since they spend 6 –8 hours daily in the dental laboratory. Also if we consider the fact that on an average a lecturer would spend at least 1 hour in the dental prosthesis laboratory, it can have detrimental effects on the hearing ability over a prolonged period.

It is therefore evident that ways and means of reducing the levels of sound in preclinical, clinical and laboratory areas should be given some thought.

## Conclusion

High levels of noise produced by various dental handpieces and equipment are potentially hazardous to personnel who work in such an environment for a prolonged period i.e, dentists and dental auxiliaries. Therefore it becomes necessary to assess the levels of noise from such equipment in a dental institution. The purpose of the present study was to fulfill this necessity. The results of the present study show that within the dental clinics, the noise levels produced by dental equipment did not exceed the permissible level of 85 dB. But the values greater than 85 dB have been recorded in the dental laboratories clearly indicating the personnel working in such conditions are at risk of developing noise- induced hearing loss (NIHL). NIHL is the most prevalent irreversible industrial disease, and noise is the biggest compensatable occupational hazard (18). As NIHL is not a treatable condition and can only be partly relieved by rehabilitative means, prevention assumes greater importance.

As part of prevention strategy, the following, recommendations are made:

Exclusion from noise exposure of those who are most susceptible to NIHL.

Reduce exposure time.

Evaluation of all personnel at regular intervals.

Regular maintenance of equipment.

Working areas to be made more acoustically satisfactory.

Personal protection with the use of ear plugs or ear muffs.

Hearing conservation programmes, including audiometry and workers’ education, should be introduced wherever needed.
